# Co‐occurrence of bobcats, coyotes, and ocelots in Texas

**DOI:** 10.1002/ece3.6242

**Published:** 2020-04-21

**Authors:** Jason V. Lombardi, Darryl I. MacKenzie, Michael E. Tewes, Humberto L. Perotto‐Baldivieso, José M. Mata, Tyler A. Campbell

**Affiliations:** ^1^ Caesar Kleberg Wildlife Research Institute Texas A&M University–Kingsville Kingsville TX USA; ^2^ Proteus Outram New Zealand; ^3^ Department of Ecosystem Science and Management College of Agriculture and Life Sciences Texas A&M University–College Station College Station TX USA; ^4^ East Foundation San Antonio TX USA

**Keywords:** *Canis latrans*, co‐occurrence, *Leopardus pardalis*, log‐linear modeling, *Lynx rufus*, multispecies occupancy models

## Abstract

Interspecific competition among carnivores has been linked to differences in behavior, morphology, and resource use. Insights into these interactions can enhance understanding of local ecological processes that can have impacts on the recovery of endangered species, such as the ocelot (*Leopardus pardalis*). Ocelots, bobcats (*Lynx rufus*), and coyotes (*Canis latrans*) share a small geographic range overlap from South Texas to south‐central Mexico but relationships among the three are poorly understood. From May 2011 to March 2018, we conducted a camera trap study to examine co‐occurrence patterns among ocelots, bobcats, and coyotes on the East Foundation's El Sauz Ranch in South Texas. We used a novel multiseason extension to multispecies occupancy models with ≥2 interacting species to conduct an exploratory analysis to examine interspecific interactions and examine the potential effects of patch‐level and landscape‐level metrics relative to the occurrence of these carnivores. We found strong evidence of seasonal mutual coexistence among all three species and observed a species‐specific seasonal trend in detection. Seasonal coexistence patterns were also explained by increasing distance from a high‐speed roadway. However, these results have important ecological implications for planning ocelot recovery in the rangelands of South Texas. This study suggests a coexistence among ocelots, bobcats, and coyotes under the environmental conditions on the El Sauz Ranch. Further research would provide a better understanding of the ecological mechanisms that facilitate coexistence within this community. As road networks in the region expand over the next few decades, large private working ranches will be needed to provide important habitat for ocelots and other carnivore species.

## Graphical abstract

This study aimed to use a novel multiseason extension to multispecies occupancy models with ≥2 interacting species to explore how ocelots, bobcats, and coyotes may potentially behave with one another (e.g., co‐existence/avoidance) and potential effects of landscape factors in the co‐occurrence of the focal species. Our study suggests a strong positive association among the focal species indicating coexistence at the temporal scale of this study. Our study has shown these mutual interactions may also have implications for planning ocelot recovery strategies in the rangelands in southern Texas.

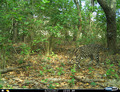

## 1 INTRODUCTION

Species interactions help shape ecological and biological functions and processes across ecosystems (Di Bitetti, Angelo, Blanco, & Paviolo, 2010). Assessments of species co‐occurrence patterns that vary in space and time are often a valuable tool in understanding the dynamics of these interactions (MacKenzie et al., 2018). Interspecific interactions such as competition, aggression, and predation and the reciprocal effects can promote or limit potential coexistence functions between different species (Davis et al., 2018; Santos et al., 2019). Taxa within Carnivora have been widely studied, given their role affecting prey populations, and subsequent habitat structure, and ecological integrity (Nagy‐Reis, Nichols, Chiarello, Ribeiro, & Setz, 2017). Examining the co‐occurrence patterns of carnivores can help identify the underlying factors affecting local species distributions, ecological functions, and partitioning of resources (Rosenzweig, 1966; Schoener, 1974; Davis et al., 2011; Davis et al., 2018).

Two or more similar‐sized species that share similar niches cannot coexist without one species being excluded from the community (Di Bitetti et al., 2010). The causative mechanism can be interference competition, where one species is directly antagonistic toward another and exploitative competition, where indirect interactions between species occur for a shared resource (Lesmeister, Nielsen, Schauber, & Hellgren, 2015). In North America, coyote (*Canis latrans*) exhibit interspecific competition and aggression toward sympatric canids (Randa & Yunger, 2006) and smaller mesocarnivores (Crooks & Soulé, 1999). In Central and South America, ocelots (*Leopardus pardalis*) negatively affect the spatial distribution of smaller felids such as southern tiger cats (*Leopardus guttulus*), and jaguarundi (*Puma yagouaroundi*), a hypothesis termed the “Pardalis Effect” (Massara et al., 2018; Nagy‐Reis et al., 2017; de Oliveira et al., 2010; Santos et al., 2019).

Unlike competitive exclusion or aggression, mutual occurrence of species is often facilitated by niche segregation (Davis et al., 2018; Di Bitetti et al., 2010; Santos et al., 2019). The ability for ≥2 species to coexist relies on differences in fitness and niche overlap, and these niches are fundamentally a function of interspecific interactions (Smith, Thomas, Levi, Wang, & Wilmers, 2018). In the case of niche segregation, these can help alleviate foraging competition and decrease potential negative effects of displacement by another species (Witczuk, Pagacz, Gliwicz, & Mills, 2005). In Belize, ocelot activity was correlated with areas of jaguar presence due to a shared preference for habitats (Davis et al., 2011). Davis et al. (2018) suggested that spatial coexistence between overlapping carnivores might be reduced through fine‐scale partitioning of activity patterns. Further, spatial coexistence can also be facilitated by human impacts and landscape‐scale features (Lesmeister et al., 2015; Smith et al., 2018).

**FIGURE 1:**
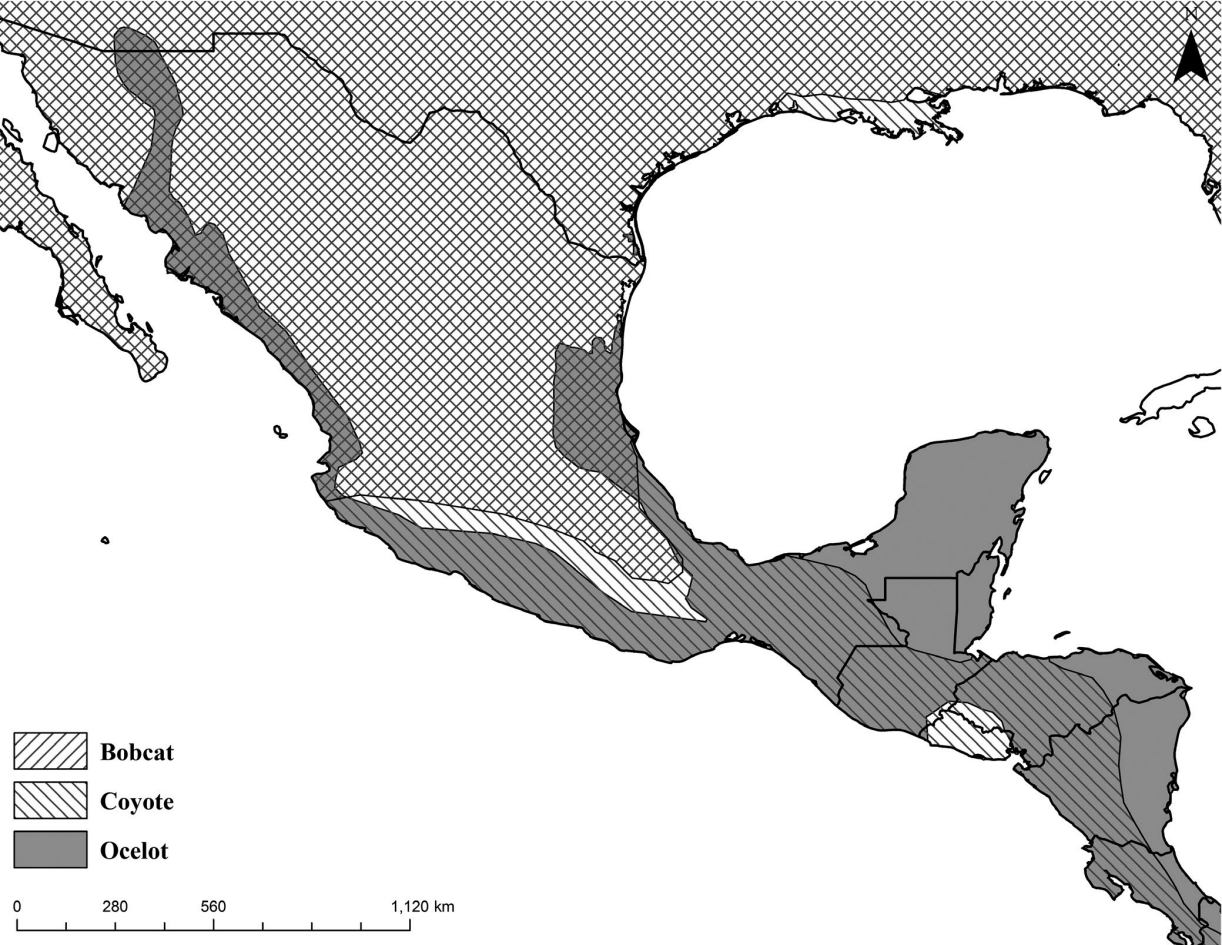
Geographic ranges and areas of geographic overlap of ocelots (*Leopardus pardalis*), bobcats (*Lynx rufus*), and coyotes (*Canis latrans*) in the southern United States, Mexico, and Central America (IUCN, 2016)

In North America, ocelots, bobcats, and coyotes share a restricted area of overlap from South Texas to south‐central Mexico (Hidalgo‐Mihart, Cantú‐Salazar, González‐Romero, & López‐González, 2004; Hody & Kays, 2018) (Figure 1). Bobcats and coyotes are abundant and sympatric across Texas. Ocelots, endangered in the United States, occur in two isolated breeding populations, the larger “Ranch population” on private working rangelands in Willacy and Kenedy counties and the “Refuge population” on protected lands in Cameron County, Texas (Tewes, 2019). Co‐occurrence patterns between sympatric bobcats and coyotes have been well‐studied (Chamberlain & Leopold, 2005; Constible, Chamberlain, & Leopold, 2006; Lesmeister et al., 2015; Smith et al., 2018); however, no study to date has examined ocelot–coyote interactions, despite sharing a more extensive geographic overlap, which extends to the Panama Canal (Hody & Kays, 2018) (Figure 1).

Studies examining bobcat–ocelot interactions in Texas seem to suggest both species mutually co‐occur in the same areas, with spatial coexistence facilitated by fine‐scale habitat partitioning. Furthermore, Leonard (2016) found that ocelots and bobcats often shared overlapping 95% home ranges and were both associated with closed‐canopy forests at the home range, with ocelots using dense canopies more than bobcats.

Using long‐term camera trap monitoring, habitat metrics, and occupancy modeling (Rota et al., 2016), we can now study the interactions (i.e., avoidance or coexistence patterns) of such unique carnivore guilds and discern potential effects of habitat variables. Such results can aid in explaining potential thresholds for occurrence, habitat use, and help guide management or recovery strategies (Crooks, 2002; Meek et al., 2014; Wang et al., 2019; Zemanova et al., 2017).

**FIGURE 2:**
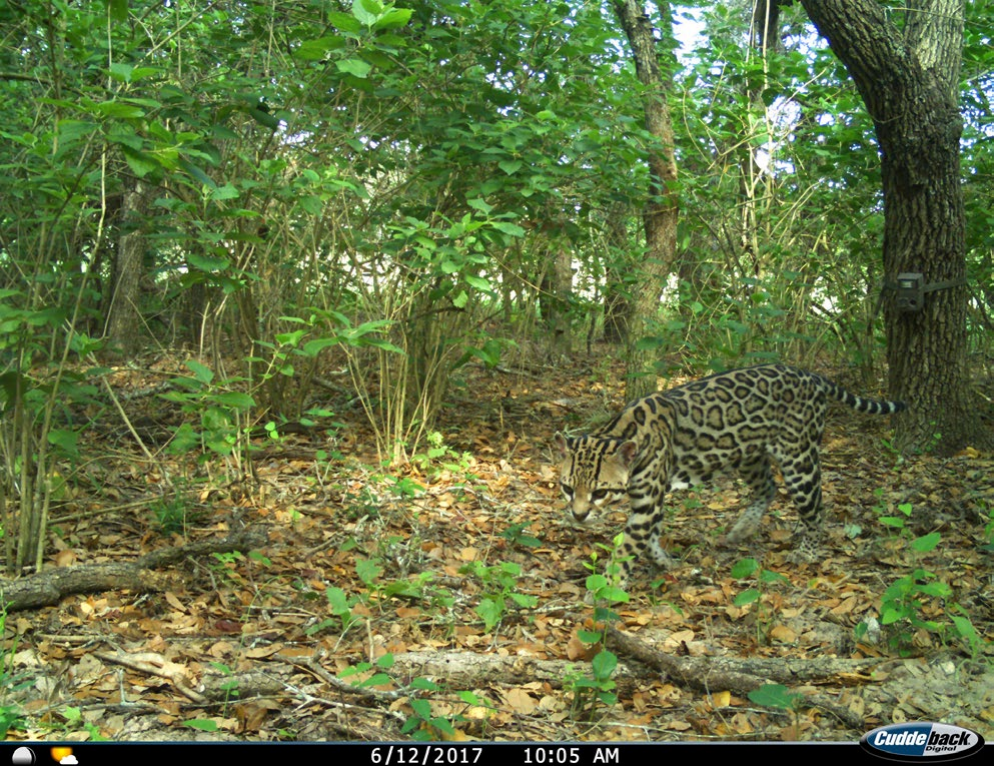
Ocelot (*Leopardus pardalis*) on in a mixed canopy live oak (*Quercus virginiana*)–American beautyberry (*Callicarpa americana*) stand on the East Foundation's El Sauz Ranch, Willacy and Kenedy counties, Texas.

From 2011 to 2018, we conducted a camera trap study in South Texas to explore ocelot–bobcat–coyote interactions and potential effects of landscape‐ and patch‐level metrics relative to the occurrence of the focal species (Figure 2). This study is the first application of a novel and multiseason extension to the multispecies occupancy model (MSOM) of two or more interacting species developed by Rota et al. (2016) using a log‐linear parameterization (MacKenzie et al., In Review). Due to the absence of predator control in the study area and surrounding ranches, we expected to observe a more natural dynamic between the species, free from man‐made influences (e.g., hunting pressure). Based on previous studies, we defined three principal hypotheses for this study: (a) probability of ocelot and bobcat occurrence and detection will be negatively influenced by the presence/detection of coyotes, but ocelot and bobcat will exhibit positive co‐occurrence values; (b) there will be season‐specific variations in detectability and occurrence of each species; (c) ocelot occurrence will be positively linked to dense canopy cover, lower woody patch density and higher forest cover, lower edge density and farther from roads; (d) bobcat occurrence will be positively linked to areas with less dense woody patches but greater edge densities, mixed canopies and more forest cover, and farther from roads; and (e) coyote occurrence will be linked to less forest cover, greater edge and patch densities, farther from roads and open canopy cover.

## 2 MATERIAL AND METHODS

### 2.1 Study area

**FIGURE 3:**
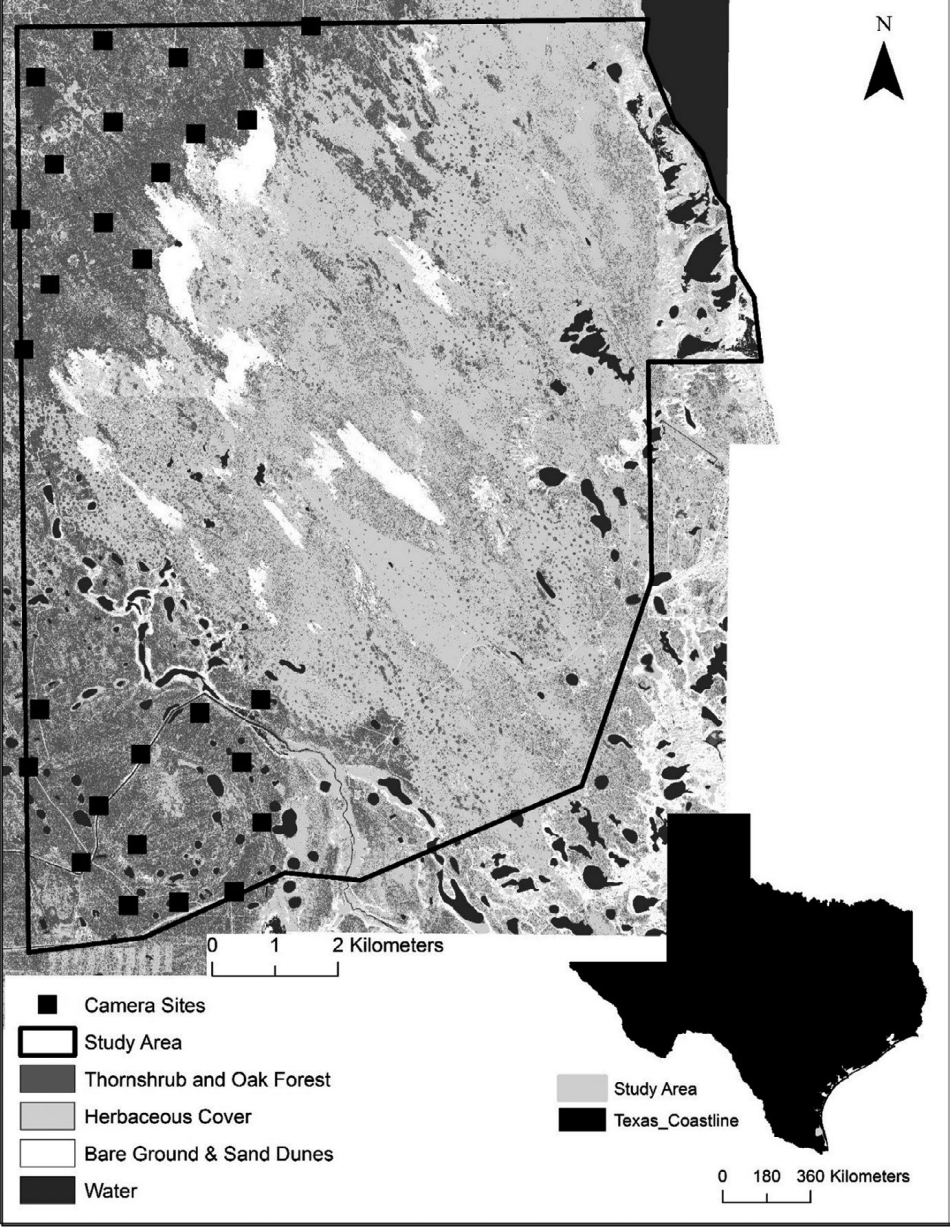
Study area and locations of 28 camera stations in the northwestern and southwestern areas of the East Foundation's El Sauz Ranch, Willacy County, Texas, USA used for camera surveys for ocelot (*Leopardus pardalis*), bobcat (*Lynx* *rufus*), and coyote (*Canis latrans*) camera surveys from 8 May 2011 to 24 March 2018.

The study was conducted on the East Foundation's El Sauz Ranch (hereafter, El Sauz) in Willacy and Kenedy counties, Texas, USA (Figure 3). This region of Texas had a semiarid subtropical climate (10–36°C) with episodic droughts (Norwine & Kuruvilla, 2007). El Sauz (113 km^2^) is managed for cattle ranching and wildlife, land stewardship conservation and was located at the comingling of the Coastal Sand Plain, Lower Rio Grande Valley, and Laguna Madre Barrier Islands and Coastal Marshes eco‐regions (Bailey & Cushwa, 1981). El Sauz, which was surrounded by other large private working rangelands, was adjacent to the Laguna Madre and the coastal town of Port Mansfield, Texas (pop. 226). The southern boundary of El Sauz was adjacent to a high‐speed roadway identified as Texas Farm‐to‐Market 186. El Sauz Ranch was composed of northwesterly parabolic inland dunes (>15 m height; Forman, Nodt, Gomez, & Pierson, 2009), lagunas and anthropogenic waterways, coastal prairie, palustrine emergent wetlands, honey mesquite (*Prosopis glandulosa*)–live oak (*Quercus virginiana*) forests, and thornscrub (e.g., lime prickly ash [*Zanthoxylum fagara*], huisache [*Acacia farnesiana*], and spiny hackberry [*Celtis pallida*]) (Shindle & Tewes, 1998; Leslie, 2016).

### 2.2 Noninvasive camera surveys

We conducted camera surveys on the El Sauz Ranch from 1 May 2011 to 31 March 2018, as a part of a long‐term ocelot‐monitoring project. Camera grids (1 × 1 km) were designed based on a systematic, grid‐based sampling method with one randomized sampling point (i.e., camera station) within each grid cell (Lombardi, Comer, Scognamillo, & Conway, 2017; Meek et al., 2014). Following United States Fish and Wildlife Service guidelines (Permit Number permit TE822908‐0) for ocelot camera surveys, we maintained a minimum of 1 km spacing between adjacent camera stations. This distance was originally defined based on mean minimum distance moved by ocelots using historic telemetry data collected in the early 2000s on a nearby private ranch. Due to previous suggesting ocelots in the region are forest‐interior species (Harveson, Tewes, Anderson, & Laack, 2004; Horne, Haines, Tewes, & Laack, 2009; Tewes, 1986), camera grid cells were established in the live oak–thornscrub forests located in southwestern (*n* = 13) and northwestern (*n* = 15) areas of the ranch. At each sampling point, camera stations were in areas within or adjacent to patches of thornscrub or live oak. At each camera station, two Cuddeback^®^ Expert Scouting Cameras and Cuddeback^®^ X‐Change Color cameras (Non‐Typical Inc) were attached to trees or wooden stakes 0.5 m above the ground. Each camera faced each other and was offset 1–2 m (Lombardi et al., 2017; Satter et al., 2019) to individually identify ocelots for the long‐term monitoring project identify individuals for the concurrent monitoring study. No bait or lure was used to avoid influencing the behavior of the focal species.

### 2.3 Environmental variables

We quantified landscape‐ or patch‐level metrics we believe likely influenced seasonal co‐occurrence patterns. To examine whether the spatial structure of woody vegetation influenced seasonal co‐occurrence patterns, we conducted a 1‐m land cover classification of the study area using 2014, 1‐m National Agriculture Imagery Program Digital Orthophoto Quarter Quadrangles (Texas Natural Resources System) in ERDAS IMAGINE (Hexagon Geospatial) based on four broad habitat categories: herbaceous (i.e., coastal prairie, herbaceous emergent wetlands, grasslands), water (i.e., lagunas and anthropogenic waterways), bare ground (inland dunes, caliche roads, and Texas Farm‐to‐Market 186 [paved road]), and woody cover (thornscrub, mesquite, and live oak forests and mottes) (Jensen, 2016; Mata et al., 2018). Using a Trimble^®^ Geo 7 Series Handheld Computer with 1 m precision or a Trimble Nomad^®^ 1050 Series Handheld Computer with GBSS 1 m precision (Trimble Navigation, Ltd), we collected 629 ground‐truth points collected in June and September 2016. We accurately assessed our classification using a confusion matric until we attained an 85% threshold (Mata et al., 2018). Because camera stations were placed 1 km apart, we placed 500 m buffers (hereafter, sampling unit) around each station, to avoid potential spatial pseudoreplication among sampling units (Lombardi et al., 2017). Within each buffer, we used FRAGSTATS 4.2 to examine three landscape metrics: woody patch density (PD; # patches/100 ha), edge density (ED; m/ha), and percent landscape (PLAND; %) (Zemanova et al., 2017). Due to previous research linking the occurrence of these species with canopy cover and distance to paved roads (Cain, Tuovila, Hewitt, & Tewes, 2003; Haines, Tewes, & Laack, 2005; Hinton, Manen, & Chamberlain, 2015; Horne et al., 2009), we attempted to examine the effect of each using a representative measurement for each sampling unit. The distance (km) from each camera station to the roadway was measured. Due to the location of the high‐speed roadway on the southern boundary of the ranch and the availability of larger forest patches farther from the road, we believe this variable may act as a proxy for greater availability of forested habitat for each species. Canopy cover was quantified using a Geographic Resource Solutions^®^ (Geographic Resource Solutions) convex densitometer at 5 m in four cardinal directions and at the center of the camera station and then averaged the five values for each station. Canopy cover estimates were categorized into three classes (open < 25%, mixed 25%–75%, and dense > 75%).

### 2.4 Multiseason multispecies occupancy models of three species

In Program R 3.6.1 (R Core Team, 2019), we implemented a novel multiseason extension (see MacKenzie et al., In Review) to the multispecies occupancy model of two or more interacting species (MacKenzie et al., 2018; Rota et al., 2016) to identify how behavior and habitat variables influence seasonal co‐occurrence patterns of ocelots, bobcats, and coyotes in South Texas. This new multiseason extension implements a multistate, multiseason modeling framework previously described by MacKenzie, Nichols, Seamans, and Gutiérrez (2009) and MacKenzie et al. (2018). A similar symmetric parameterization of multispecies occupancy models with ≥2 interacting species was used, where the effects were mutual for each species (MacKenzie et al., 2018; Rota et al., 2016). Unlike other multispecies models (see Richmond, Hines, & Beissinger, 2010; Walls, Waddle, & Dorazio, 2011), species were not considered dominant or subordinate to each other. Here, each state was a combination of presence/absence of each species; therefore, the multinomial probabilities could be modeled using indicator variables for each species in combination with the multinomial logit‐link function. When there are ≥3 species, there is the potential for higher‐order interactions, which may be difficult to interpret or estimate with small sample sizes; however, the higher‐order interactions do not have to be estimated (i.e., independence is assumed among the group of species at that level) (MacKenzie et al., In Review).

For this study, we defined a capture history containing 14 seasons with five monthly (4‐week) survey occasions per season (i.e., each season was 20 weeks, with five surveys). Seasons were partitioned based on average temperatures over the sampling period (i.e., cool [18.4°C]: 8 November to 24 March; hot [29°C]: 8 May to 23 September). A 4‐week survey occasion period was chosen to avoid violating the assumption of independent detection for the coyote and bobcat datasets. A species was classified as detected during a survey occasion if it was photographed at least once during that period. We implemented a non‐Markovian multiseason model, where the probability of occupancy is independent of the previous occupancy state of a unit, which allows for season‐specific occupancy probabilities (MacKenzie et al., 2018). A non‐Markovian model was assumed to reduce the number of parameters to estimate due to the statistically small size.

Two small sets of candidate models were considered based on biological relevant a priori hypotheses regarding the co‐occurrence patterns of ocelots, bobcats, and coyotes. Each candidate model set was analyzed separately to examine both behavioral influences on co‐occurrence and the potential effects of habitat metrics. The first set of candidate models (*H*_1_‐*H*_5_, plus a null model) were based on five a priori hypotheses examining the influence of behavior on detection and occupancy (Table A1). We hypothesized that the likelihood of felid occurrence (ocelots and bobcats) will be negatively influenced by the presence of coyotes (Hunter, 2019; Neale & Sacks, 2001). However, based on previous studies of ocelot–bobcat interactions in Texas (Horne et al., 2009; Leonard, 2016), we believed ocelots and bobcats would exhibit positive co‐occurrence values. We assumed a species‐specific effect on detection (Model *H*_1_) and season effect on detection (Model *H*_2_). Models *H*_3_ and *H*_4_ reflected the hypothesis that detectability of ocelots and bobcats was negatively affected by the presence of coyotes in each occasion, respectively. Model *H*_5_ refers to the hypothesis that occupancy of all species varied seasonally, and detection was a function of a species‐specific seasonal effect. A null model (*H*_6_) with no species interaction or seasonal effects on occupancy and detection was also considered.

Our second set of candidate models (*H*_7_‐*H*_11_) examined the potential effects of landscape‐ and patch‐level variables on occurrence of each species (Table A1). We did not consider models that failed to converge, as this may be a result of over‐parameterization for the sample size, or it is just a bad likelihood function with multiple maxima. Due to the complexity of these models, we limited models that tested the effects of these variables to no more than two biologically relevant covariates. Canopy cover around each sampling unit was used as a categorical variable where mixed canopies were used as a reference level as it was the most dominant cover type in the study area. Based on Horne et al. (2009) and Andelt (1985), Model *H*_7_ reflected the hypothesis that compared to mixed cover, ocelots are more likely to occur in dense canopies, bobcats were negatively affected by dense cover, but positively respond to mixed and open cover types, and coyotes were positively influenced by open canopies. Due to the presence of a high‐speed roadway on the southern boundary of the ranch, and the known impact of roads on ocelots (Haines et al., 2005), bobcats (Cain et al., 2003), and coyotes (Hinton et al., 2015), we hypothesized that proximity to roads affected felids and coyotes (Model *H*_8_). Past studies have illustrated the importance of incorporating landscape metrics in discerning effects on the occurrence and habitat use of the focal species (Jackson, Laack, & Zimmerman, 2005; Neale & Sacks, 2001; Randa & Yunger, 2006). As such, three hypotheses (Models *H*_9_ *‐H*_11_) were developed to test: (1) ocelot and bobcat occurrence positively influenced by to areas of low woody patch density, while coyote occurrence is lower to areas of high patch density; (2) ocelots and bobcats are more likely than coyotes to occur in areas with a greater percentage of woody cover; and (3) bobcats and coyotes will be more likely to occur in areas with a greater edge density (per 100 ha) than ocelots which will be more likely to occur in areas with a lower edge density.

Parameter estimates for each hypothesis were estimated using 85% confidence intervals (CIs) (Arnold, 2010). We compared each set of candidate models with Akaike's information criterion (AIC) in R 3.6.1 (R Core Team, 2019) using the difference in AIC to determine which model best explained each candidate model selection.

## 3 RESULTS

**FIGURE 4:**
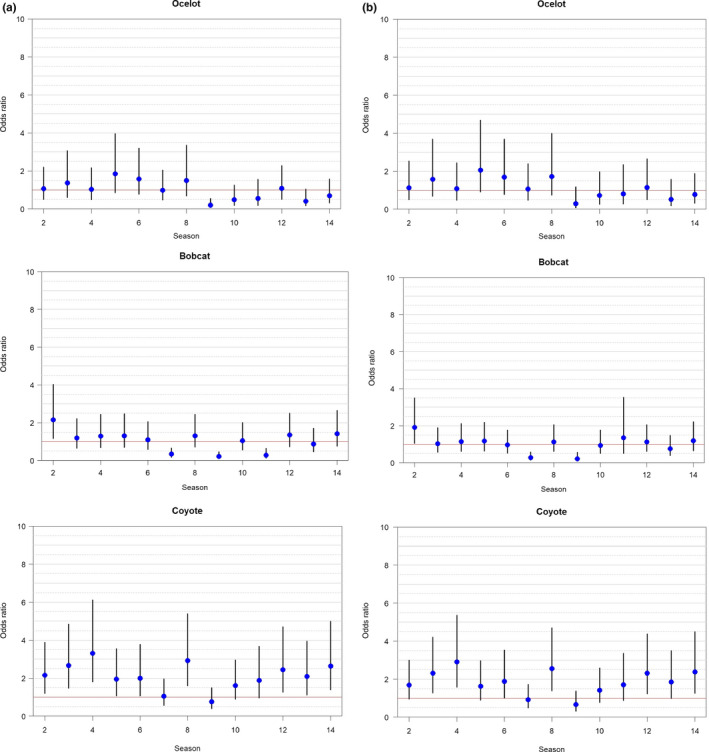
Odds ratio with 95% CI of predicted seasonal detection for ocelot (*Leopardus pardalis*), bobcat (*Lynx rufus*), and coyote (*Canis latrans*) for a seasonal interaction model (a) and a seasonal distance to high‐speed roadway (km) model (b) from 8 May 2011 to 24 March 2018 on the East Foundation's El Sauz Ranch, Willacy and Kenedy counties, Texas, USA

**FIGURE 5:**
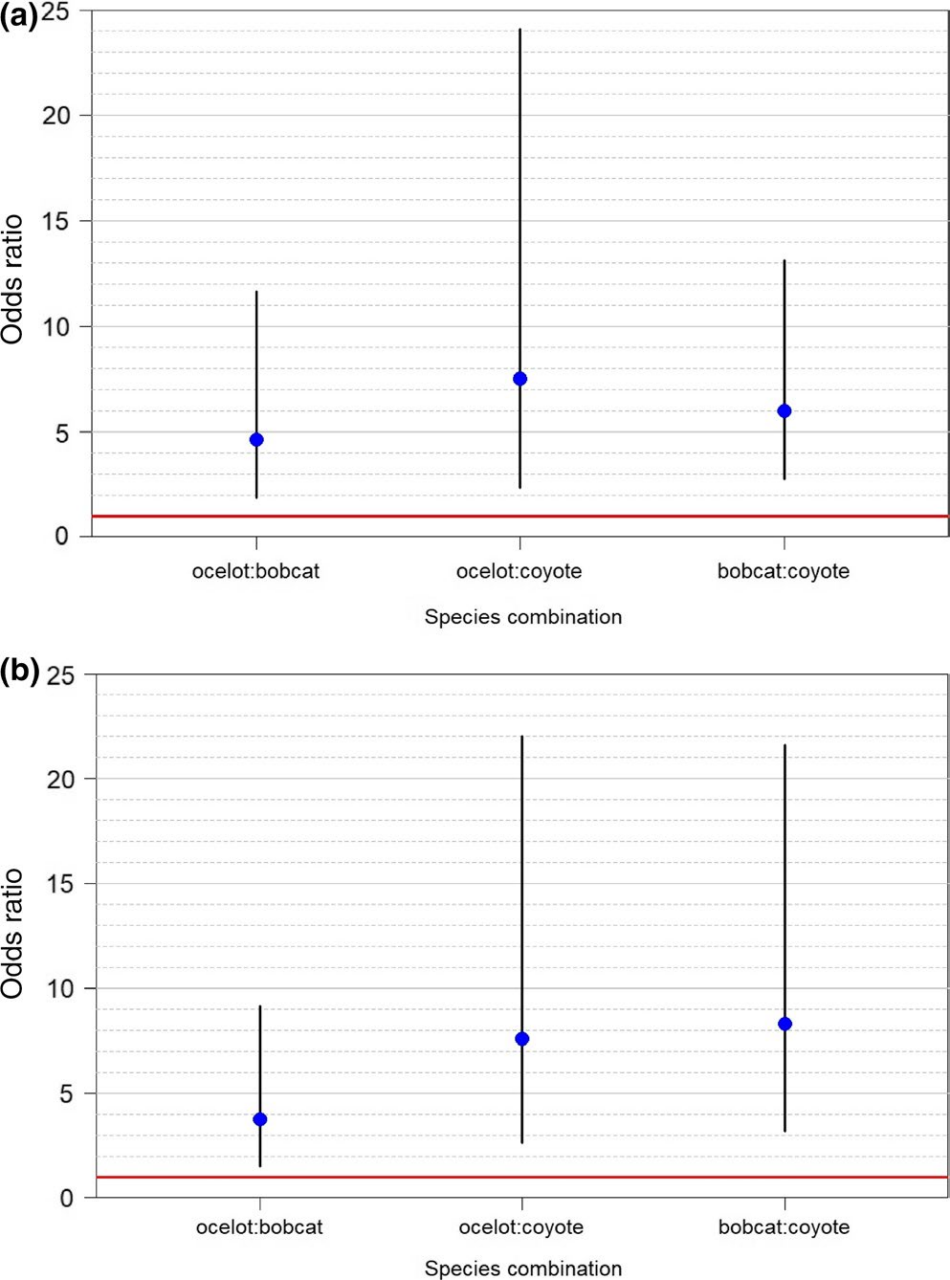
Odds ratio with 95% CI of predicted presence of ocelot (*Leopardus pardalis*):bobcat *(Lynx rufus*), ocelot:coyote (*Canis latrans*) and bobcat:coyote co‐occurrence based on a seasonal interaction model (A) and seasonal distance to road model (B) from 8 May 2011 to 24 March 2018 on the East Foundation’s El Sauz Ranch, Willacy County, Texas, USA

**FIGURE 6:**
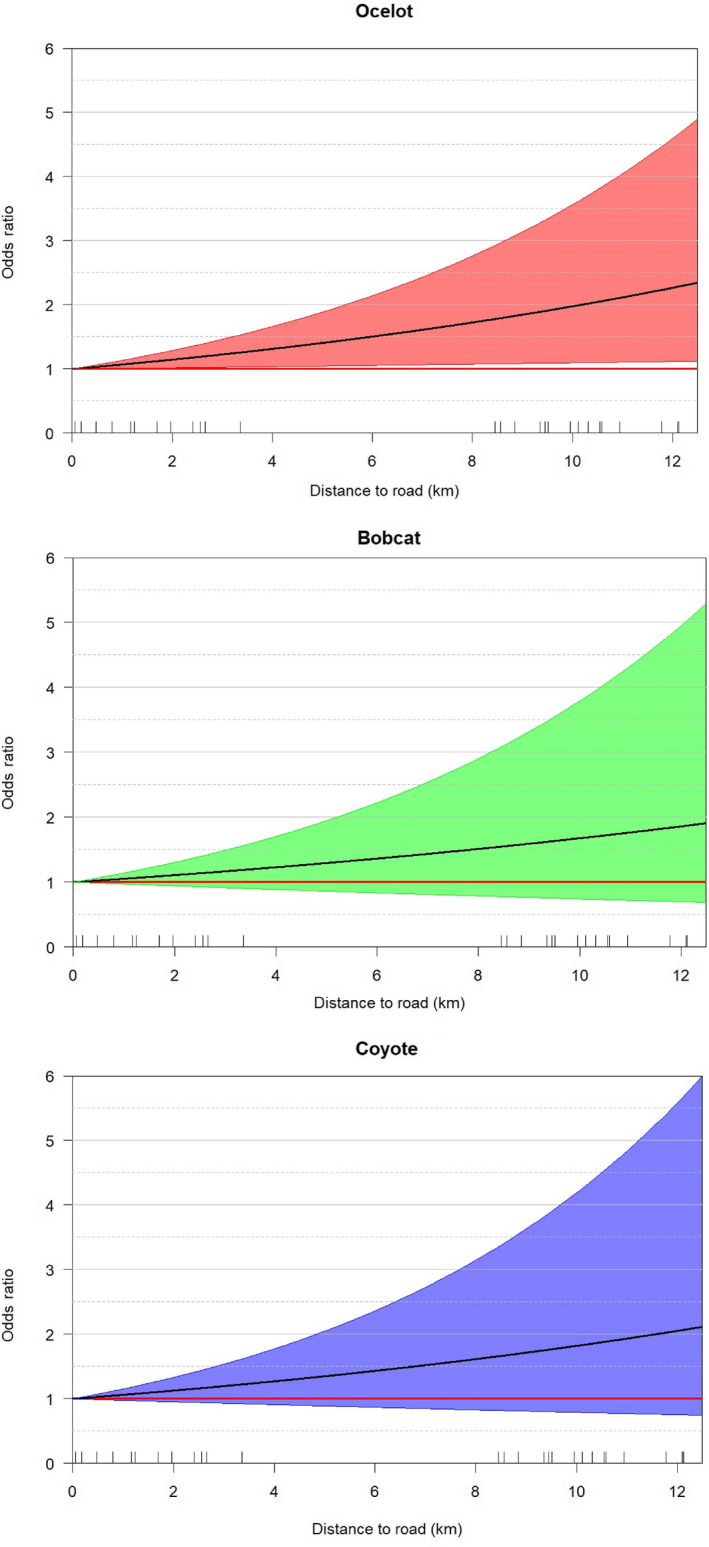
Odds ratio with 95% CI of the predicted presence of ocelot (*Leopardus pardalis*), bobcat (*Lynx rufus*), and coyote (*Canis latrans*) relative to the distance to high‐speed roadway (km) from 8 May 2011 to 24 March 2018 on the East Foundation's El Sauz Ranch, Willacy and Kenedy counties, Texas, USA

Over 250,000 photographs were recorded over 3,920 trap months from 2011 to 2018. Of the three species, we documented >2,000 coyote detections, 1,529 bobcat detections, and 1,076 ocelot detections (Table A2). Camera stations on the ranch occurred within 55.1% woody cover, of which 60.7% contained mixed woody canopies and 32.1% dense woody canopies (Table 1). For our first model set, we found that coyotes did not negatively influence ocelots or bobcats, rather each species mutually co‐occurred in the study area. Positive effects among across all pairwise species interactions were observed (ocelot–bobcat [*β* = 1.53, CI: 1.04–2.02]; ocelot–coyote [*β* = 2.02, CI: 1.40–2.63]; bobcat–coyote [*β* = 1.78, CI: 1.37–2.20]) (Table 2). The greatest real probability of occupancy was observed when all three species were present (Ψ = 0.43 [0.38–0.49]) and lowest probability with only ocelot present at a site (Ψ = 0.005[0.002–0.01]) (Table 3). Detection was best explained by a species‐specific seasonal trend (Figure 4) and not by interactions with other species. The odd of ocelots occurring in a cell was estimated to be 4–5 times higher in areas with bobcats (and vice versa) (Figure 5). For ocelots and bobcats, the odds of occupancy were 6–7 times greater in areas with coyotes, and the likelihood of all three co‐occurring was cumulative at the probability scale (Figure 5). For our habitat models, we assumed detection was a function of a species‐specific seasonal trend since detection was not influenced by interacting species based on models *H*_3_ and *H*_4_. Proximity to the high‐speed roadway best explained the effect of habitat on the co‐occurrence of ocelots, bobcats, and coyotes (Table 4). Increasing distance (km) from the highway had a positive effect on the occurrence of ocelots (*β* = 0.07, CI: 0.04–0.10), bobcats (*β* = 0.07, CI: 0.02–0.11), and coyotes (*β* = 0.06, CI: 0.02–0.10) (Figure 6).

**TABLE 1:** Habitat variables measured at each camera station used for species co‐occurrence study of bobcats (*Lynx rufus*), coyotes (*Canis latrans*), and ocelots (*Leopardus pardalis*) on the East Foundation's El Sauz Ranch, Kenedy and Willacy counties, Texas, 8 May 2011–24 March 2018

Variable	$\bar{x}$ (*SD*)
Distance to paved road (km)	6.46 (4.37)
Woody patch density (# patches/100 ha)	443.95 (194.16)
Edge density (m/100 ha)	1,151.1 (146.1)
Open canopy cover (% of stations)	7.15
Mixed canopy cover (% of stations)	60.71
Closed‐canopy cover (% of stations)	32.14
Mean canopy cover (%)	56.94 (22.36)

**TABLE 2:** Model selection results for candidate set 1 (interspecific interactions) for multiseason multispecies occupancy analyses used to estimate co‐occurrence (ψ) and detection (p) of ocelot (*Leopardus pardalis*), bobcat (*Lynx rufus*), and coyote (*Canis latrans*) on the East Foundation's El Sauz Ranch, Willacy and Kenedy counties, Texas from 8 May 2011 to 24 March 2018

Model	AIC	dAIC	*w*	*K*
ψ (spA + spB + spC + spA:spB + spA:spC + spB:spC, p (DspA + DspB + DspC + DspA:SEAS + DspB:SEAS + DspC:SEAS)	6,125.42	0.00	100.00	48
ψ (spA + spB + spC + SEAS), p (DspA + DspB + DspC + DspA:SEAS + DspB:SEAS + DspC:SEAS)	6,172.16	46.75	0.00	58
ψ (spA + spB + spC), p (DspA + DspB + DspC + OspB:OspA:DspB + OspB:OspC:DspB)	6,185.41	59.99	0.00	8
ψ (spA + spB + spC + spA:spB + spA:spC + spB:spC), p (DspA + DspB + DspC)	6,201.06	75.64	0.00	9
ψ (spA + spB + spC), p (DspA + DspB + DspC + OspA:OspB:DspA + OspA:OspC:DspA)	6,251.29	125.87	0.00	8
ψ (spA + spB + spC), p (DspA + DspB + DspC)	6,310.15	184.73	0.00	6

Note

Models with a difference in AIC < 2.00 are most plausible, with associated model weight (*w*) and number of parameters (*K*).

SpA refers to ocelots, spB refers to bobcat, and spC refers to coyotes; DspABC refers to detection of species A, B, or C; OspABC refers to the presence of species A, B, or C; and SEAS refers to seasonal effect.

**TABLE 3:** Estimated mean occupancy probabilities for each occupancy state for ocelots ((*Leopardus pardalis;* Species A), bobcats (*Lynx rufus;* Species B), and coyotes (*Canis latrans;* Species C) on the El Sauz Ranch, Willacy and Kenedy counties, Texas

State	Est	*SE*
abc	0.115	0.018
Abc	0.005	0.004
aBc	0.064	0.015
ABc	0.014	0.008
abC	0.078	0.018
AbC	0.028	0.011
aBC	0.261	0.028
ABC	0.433	0.030

**TABLE 4:** Model selection results for candidate set 2 (habitat effects) for multiseason multispecies occupancy analyses used to estimate co‐occurrence (ψ) and detection (p) of ocelot (*Leopardus pardalis*), bobcat (*Lynx rufus*), and coyote (*Canis latrans*) on the East Foundation's El Sauz Ranch, Willacy and Kenedy counties, Texas, from 8 May 2011 to 24 March 2018

Model	AIC	dAIC	*w*	*K*
ψ (spA + spB + spC + spA:spB + spA:spC + spB:spC + spA:SEAS + spB:SEAS + spC:SEAS + DistRoad:(spA + spB +spC), p (DspA + DspB + DspC + DspA:SEAS + DspB:SEAS + DspC:SEAS)	6,119.75	0.00	0.797	90
ψ (spA + spB + spC + spA:spB + spA:spC + spB:spC + spA:SEAS + spB:SEAS + spC:SEAS + WPD:(spA + spB +spC), p (DspA + DspB + DspC + DspA:SEAS + DspB:SEAS + DspC:SEAS)	6,122.65	2.89	0.187	90
ψ (spA + spB + spC + spA:spB + spA:spC + spB:spC + spA:SEAS + spB:SEAS + spC:SEAS + WPLAN:(spA + spB +spC)), p (DspA + DspB + DspC + DspA:SEAS + DspB:SEAS + DspC:SEAS)	6,128.93	9.18	0.008	90
ψ (spA + spB + spC + SEAS +Open:(spA + spB +spC)+ Dense:(spA + spB +spC), p (DspA + DspB + DspC + DspA:SEAS + DspB:SEAS + DspC:SEAS)	6,129.94	10.19	0.005	93
ψ (spA + spB + spC + spA:spB + spA:spC + spB:spC + spA:SEAS + spB:SEAS + spC:SEAS + WED:(spA + spB +spC), p (DspA + DspB + DspC + DspA:SEAS + DspB:SEAS + DspC:SEAS)	6,137.84	18.09	0.003	90

Note

Models with a difference in AIC < 2.00 are most plausible, with associated model weight (*w*) and the number of parameters (*K*).

SpA refers to ocelots, spB refers to bobcats, and spC refers to coyotes; DspABC refers to detection of species A, B, or C; SEAS refers to the seasonal effect; DistRoad refers to linear distance (km) from each camera station to Farm‐to‐Market State Highway 186; WPD refers to woody patch density (number of patches/100 ha) and WPLAN refers to percent of woody cover within our 500 m buffered sampling unit; Open (<25%) and Dense (>75%) refers to a classification of canopy cover measured within each sampling unit near the sampling location (i.e., camera station); and WED refers to the total length (m) of edge in woody patches per hectare within the 500 m buffered sampling unit.

## 4 DISCUSSION

On working rangelands free from predator control, we found that ocelots, bobcats, and coyotes did not exhibit avoidance behavior and had a greater likelihood of occurrence when the other species was also present. This was the first study to examine the interactions within this unique carnivore community across a small overlapping geographic range. Ecological research throughout the Americas has focused on ocelot interactions with other neotropical felids, but studies of interactions with bobcats or other carnivores have been limited (Horne et al., 2009; Leonard, 2016; Massara, Paschoal, Bailey, Doherty, & Chiarello, 2016; Sánchez‐Cordero et al., 2008). We were able to discern potential negative effects of distance to paved roads on the occurrence of these species, which will have implications for managing bobcat and coyote populations and recovering ocelot populations on working rangelands, especially those located adjacent to expanding urban areas. This research also highlights the ecological application of a multiseason extension to multispecies occupancy models with ≥2 interacting species use a log‐linear parameterization.

No evidence of ocelots and bobcats exhibiting negative interactions was observed on El Sauz, despite sharing a similarity in body size overlap and diet in South Texas (Booth‐Binczik et al., 2013; Schmidly & Bradley, 2016). The likelihood of ocelot occurrence was five times greater when bobcats were present. In Central and South America, ocelots exhibit top‐down forces on other small felids and small carnivores, leading to spatial avoidance, predation, and temporal segregation (Nagy‐Reis et al., 2017; de Oliveira et al., 2010). Further, it had been suggested that ocelots might limit the geographical distribution of bobcats in areas of at the southern periphery of bobcat geographic range where the two species co‐occur (Sánchez‐Cordero et al., 2008). However, due to declining populations of ocelots in the United States, Horne et al. (2009) suggested larger densities of bobcats may negatively influence ocelot occurrence. The co‐occurrence of bobcats and ocelots in smaller dense patches of thornscrub was the result of fine‐scale resource partitioning (Horne et al., 2009). Although we did not observe effects of habitat variables between bobcats and ocelots, Leonard (2016) indicated these felids may exhibit temporal segregation within the study area, which may help facilitate co‐occurrence in this heterogeneous woody landscape.

Presence of coyotes was a positive indicator of bobcat and ocelot occurrence—where the likelihood for each felid was greater (6–7.5 fold) when coyotes were also present. The positive effects were likely due to an abundance of preferred cover, high availably of food resources, and olfactory cues, which would allow the three species to coexist in the same areas, despite sharing a considerable overlap in body size and trophic level. Coyote interactions with felids have been studied across their range with mixed results regarding potential negative effects such as interference competition, avoidance, predation, and aggression (Neale & Sacks, 2001; O'Donoghue, Boutin, Krebs, Murray, & Hofer, 1998; Logan & Sweanor, 2001). Hunter (2019) suggested coyotes serve as a potential predator for ocelots across their shared geographic range from South Texas to Panama (Hidalgo‐Mihart et al., 2004; Hody & Kays, 2018; Schmidly & Bradley, 2016). We did not find evidence of ocelots avoiding areas where coyotes were present. In many studies within the United States, bobcats and coyotes often shared space and bobcats did not exhibit spatial or temporal partitioning (Neale & Sacks, 2001; Thornton, Sunquist, & Main, 2004; Melville et al., 2015; Lesmeister et al., 2015). Thornton et al. (2004) suggested that reduced agonistic encounters between these species might be attributed to nonoverlapping core areas, even in areas where the two species do not segregate at the home range scale. Only two other studies examined ocelot–canid (i.e., Crab‐eating fox [*Cerdocyon thous*] and domestic dogs [*Canis lupus familiairis*]) interactions in Brazil and found no effect of avoidance by foxes (Massara et al., 2016), but a weak negative effect of free‐ranging dogs (Massara et al., 2018). Davis et al. (2011) also indicated ocelots can co‐occur in areas with other carnivores including those potentially expressing interspecific aggression such as jaguars (*Panthera onca*). Further, coyotes are more likely to exhibit interspecific aggression toward other canids (e.g., foxes) (Randa & Yunger, 2006) due to their high dietary overlap.

In South Texas, these carnivores may feed on similar prey species where they co‐occur (Andelt, 1985; Booth‐Binczik et al., 2013). Although we did not examine the effect of prey abundance on the occurrence of these carnivores, prey availability may also explain species coexistence in the study area. Native wildlife was not harvested and there is low habitat manipulation, which may help facilitate increased food availability on the ranch. Witmer and deCalesta (1986) suggested little competition between coyotes and bobcats occurring in areas with moderate prey populations or greater variety in food items for coyotes (Andelt, 1985).

The presence of high‐speed roadways adjacent to large private working ranches may affect the occurrence of medium‐sized carnivores in South Texas. These results support our hypothesis that ocelots bobcats and coyotes occur farther from roadways. Prior to this study, multiple studies have shown the negative impact of high‐speed roadways on carnivore populations in urban and wildland areas (Cain et al., 2003; Haines, Janečka, Tewes, Grassman, & Morton, 2006; Klar et al., 2008; Litvaitis et al., 2015). High‐speed roads adversely impact wildlife species by fragmenting habitats and populations and causing vehicle‐attributed mortalities, which often lead to decreased gene flow and population declines (Cain et al., 2003; Forman et al., 2003). High‐speed roadways affect the distribution and movements of wide‐ranging felids including mountain lion (*Puma concolor*), European wildcat (*Felis silvestris*), and bobcat (Dickson & Beier, 2002; Tigas et al., 2002; Cain et al., 2003; Klar et al., 2008). Further, Klar et al. (2008) reported that European wildcats generally avoid areas within 200 m of roadways.

For ocelots, paved roads are strongly associated with sources of mortality in the Lower Rio Grande Valley (LRGV) and vehicle collisions remain a major mortality factor in South Texas (Haines et al., 2005). On our study site, ocelots were detected at camera stations closest to the highway, but these dense thornscrub patches along the roadway in these areas were remnants of a larger patch of thornscrub that were cleared for brush management > 35 years ago (J. Lombardi, unpub. data). High‐speed roadways are also an important source of mortality for bobcats and coyotes across the country (Tigas et al. 2002; Litvaitis et al., 2015). In wildland and urban areas, bobcats avoid areas near roads (Tigas et al., 2002; Litvaitis et al., 2015). Litvaitis et al. (2015) suggested that bobcats may avoid roads because of perceived risk or limited prey in wild and urban areas of New Hampshire. Coyotes and bobcats occurring on South Texas working ranches use ranch roads as travel corridors (Bradley & Farge, 1988), but information regarding the use of these secondary roads intersecting with high‐speed roadways was not reported. Hinton et al. (2015) reported that resident coyotes in eastern North Carolina significantly avoided roads.

An alternate explanation is that larger patches of woody cover that are less fragmented occur farther from the highway. These patches may provide a more suitable habitat for these species located further from the high‐speed roadway. Due to the potential for higher‐order interactions, sample sizes commonly found in camera trapping studies may not provide enough data. For this reason, we purposely constructed models with single covariate for each species effect to ensure model convergence given the sample sizes observed in this study. Future research should focus on potential space use of ocelots, bobcats, and coyotes in relation to roads to understand the ecological mechanisms causing these species to occur in areas away from paved roads on the northern periphery of shared geographic overlap.

Detection was not influenced by positive species associations. The importance of olfactory marking as an intra‐ and interspecific communication mechanism among mammalian carnivores likely plays a role in this (Allen, Wallace, & Wilmers, 2015; King, Salom‐Pérez, Shipley, Quigley, & Thornton, 2017). Olfactory cues (e.g., latrines, urine, and scat) used by carnivores are usually used to indicate reproductive status, territory marking or warn individuals of their presence (Allen et al., 2015; King et al., 2017). However, it has been suggested that community scrapes and latrines may help reduce aggression and promote tolerance of neighboring individuals from the same or different species (King et al., 2017). It is plausible that while presence of such community scrapes and latrines may allow the focal species to coexist within the same areas, it may also play a role in us being unable to discern the effects of species associations in detection.

Over 7 years, the probabilities of detection between ocelots, bobcats, and coyotes varied greatly compared to the first survey season. Coyotes had the greatest probability of detection in the study area, with odds of 1.5–4 times greater compared to the first survey season. Initially, coyotes can be wary of novel objects (i.e., camera traps) in their territories, which may explain why detections increased in subsequent years (Lombardi et al., 2017). Furthermore, the social behavior of coyotes which form packs of 2–6 individuals in South Texas (Andelt, 1985), compared to the solitary nature of ocelots and bobcats may explain greater coyote detection probabilities. Unlike bobcats and coyotes, we observed a noticeable drop in odds (<1) of ocelot detections after season 8 (~2015), a drop that may indicate a loss of individuals in the area.

As urbanization and road networks in the adjacent LRGV increase over the next three decades, large private working ranches like our study area will provide important habitat for ocelots and other carnivore species (Lombardi, Perotto‐Baldivieso, & Tewes, 2020). The use of multiseason, multispecies models with two or more interacting species gives biologists and wildlife managers the ability to conduct long‐term analyses of interspecific interactions of endangered species, potential competitors, prey species, or economically valuable species. However, as the number of interacting species increases, so does the complexity of the modeling, requiring a skilled analyst to properly model and interpret the potential effects with multiple habitat covariates. The data requirements for such complex models should also be considered before commencing fieldwork to ensure sample sizes will be adequate.

Despite the absence of a larger carnivore, and perceived larger coyote and bobcat populations, ocelots do not appear to be affected by coyote and bobcat presence, which will help guide recovery efforts in areas in which all three species co‐occur. However, we acknowledge the temporal scale at which we conducted this study may have been too broad to discern more fine‐scale temporal dynamics not observed in this study. Further research should examine macro‐ and fine‐scale space use using GPS data, dietary analyses, and temporal segregation among these carnivores to discern any underlying effects not observed in this study.

## CONFLICTS OF INTEREST

None declared.

## AUTHOR CONTRIBUTIONS

**Jason V. Lombardi:** Conceptualization (equal); data curation (lead); formal analysis (lead); investigation (equal); Methodology (equal); validation (equal); visualization (equal); writing – original draft (lead); writing – review and editing (lead). **Darryl I. MacKenzie:** Conceptualization (equal); data curation (equal); formal analysis (lead); investigation (equal); methodology (equal); software (lead); validation (equal); visualization (equal); writing – original draft (supporting); writing – review and editing (supporting). **Michael E. Tewes:** Conceptualization (equal); methodology (equal); project administration (lead); resources (equal); supervision (lead); writing – original draft (supporting); writing – review and editing (supporting). **Humberto L. Perotto‐Baldivieso:** Data curation (supporting); formal analysis (equal); methodology (equal); supervision (equal); writing – original draft (supporting); writing – review and editing (supporting). **Jose M. Mata:** Data curation (equal); formal analysis (equal); investigation (equal); methodology (equal); validation (equal); writing – original draft (supporting); writing – review and editing (supporting). **Tyler A. Campbell:** Project administration (equal); resources (equal); writing – original draft (supporting); writing – review and editing (supporting).

## Data Availability

Data used in this manuscript (i.e., three‐species camera data, and high‐resolution spatial data) are accessible in the repository Dryad. Please see https://doi.org/10.5061/dryad.931zcrjgp.

## APPENDIX A

**TABLE A1:** List of a priori hypotheses for each candidate model set and the attributed occupancy model used to test each individual or group of hypotheses to examine potential behavioral effects of bobcats (*Lynx rufus*), coyotes (*Canis latrans*), and ocelots (*Leopardus pardalis*) interactions and effects of covariates on occurrence in Willacy and Kenedy counties, Texas, USA from 2011 to 2018

Model Set	A priori hypotheses	Specified model to test for each set of hypothesis
1	Probability of felid occurrence (ocelots and bobcats) will be negatively influenced by the presence of coyotes. (2) The probability of felid co‐occurrence will be positively influenced by each other. (3) Detectability will be influenced by a seasonal trend for each species	psi = ~spA + spB + spC + spA:spB + spA:spC + spB:spC, p = ~DspA + DspB + DspC
		psi = ~spA + spB + spC + spA:spB + spA:spC + spB:spC, p = ~DspA + DspB + DspC + DspA:SEAS + DspB:SEAS + DspC:SEAS
	Detectability of felids will be negatively influenced by coyote presence, but positively influenced by other felids	psi = ~spA + spB + spC, p = ~DspA + DspB + DspC + OspB:OspA:DspB + OspB:OspC:DspB
		psi = ~spA + spB + spC, p = ~DspA + DspB + DspC + OspA:OspB:DspA + OspA:OspC:DspA
	We will observe a species‐specific seasonal trend in occupancy and detection for each species	psi = ~spA + spB + spC + SEAS, p = ~DspA + DspB + DspC + DspA:SEAS + DspB:SEAS + DspC:SEAS
2	Compared to mixed cover, ocelots are more likely to occur in dense canopies, bobcats were negatively affected by dense cover, but positively respond to mixed and open cover types, and coyotes were positively influenced by open canopies	psi = ~spA + spB + spC + SEAS +Open:(spA + spB +spC)+ Mix:(spA + spB +spC), p = ~DspA + DspB + DspC + DspA:SEAS + DspB:SEAS + DspC:SEAS
	Ocelot and bobcat occurrence positively influenced by to areas of low woody patch density, while coyote occurrence is lower to areas of high patch density	psi = ~spA + spB + spC + spA:spB + spA:spC + spB:spC + spA:SEAS + spB:SEAS + spC:SEAS + WPD:(spA + spB +spC), p = ~DspA + DspB + DspC + DspA:SEAS + DspB:SEAS + DspC:SEAS
	Ocelots and bobcats are more likely than coyotes to occur in areas with a greater percentage of woody cover	psi = ~spA + spB + spC + spA:spB + spA:spC + spB:spC + spA:SEAS + spB:SEAS + spC:SEAS + WPLAN:(spA + spB +spC), p = ~DspA + DspB + DspC + DspA:SEAS + DspB:SEAS + DspC:SEAS
	Bobcats and coyotes will be more likely to occur in areas with a greater edge density (per 100 ha) than ocelots which will be more likely to occur in areas with a lower edge density	psi = ~spA + spB + spC + spA:spB + spA:spC + spB:spC + spA:SEAS + spB:SEAS + spC:SEAS + WED:(spA + spB +spC), p = ~DspA + DspB + DspC + DspA:SEAS + DspB:SEAS + DspC:SEAS
	Ocelots, bobcats, and coyote occurrence would be linked to areas farther from high‐speed roadways	psi = ~spA + spB + spC + spA:spB + spA:spC + spB:spC + spA:SEAS + spB:SEAS + spC:SEAS, + DistRoad:(spA + spB +spC), p = ~DspA + DspB + DspC + DspA:SEAS + DspB:SEAS + DspC:SEAS

**TABLE A2:** The number of detection events for each focal species (i.e., bobcats [*Lynx rufus*], coyotes [*Canis latrans*], and ocelots [*Leopardus pardalis*]) within each season (4‐week period) across 28 camera trap sites on the East Foundation's El Sauz Ranch from 2011 to 2018

	Bobcat	Coyote	Ocelot
Season 1	41	32	27
Season 2	72	68	37
Season 3	59	66	27
Season 4	57	71	31
Season 5	59	66	36
Season 6	54	57	46
Season 7	30	44	33
Season 8	54	64	31
Season 9	23	35	14
Season 10	40	46	13
Season 11	25	49	14
Season 12	57	51	30
Season 13	42	54	11
Season 14	55	52	20
